# Temporal Dispersion and Duration of the Distal Compound Muscle Action Potential Do Not Distinguish Diabetic Sensorimotor Polyneuropathy From Chronic Inflammatory Demyelinating Polyneuropathy

**DOI:** 10.3389/fneur.2022.872762

**Published:** 2022-04-26

**Authors:** Monica Alcantara, Mylan Ngo, James de la Cruz, Deepak Menon, Carolina Barnett-Tapia, Hans Katzberg, Vera Bril

**Affiliations:** ^1^Ellen and Martin Prosserman Centre for Neuromuscular Diseases, Toronto General Hospital, University Health Network (UHN), Toronto, ON, Canada; ^2^Department of Neurology, National Institute of Mental Health and Neurosciences (NIMHANS), Bangalore, India

**Keywords:** CIDP, DSP, diabetes mellitus, polyneuropathy, chronic inflammatory demyelinating polyneuropathy, nerve conduction studies

## Abstract

**Objective:**

To investigate the contribution of duration and temporal dispersion (TD) of the distal compound muscle action potential (CMAP) in discriminating chronic inflammatory demyelinating polyneuropathy (CIDP) from diabetic sensorimotor polyneuropathy (DSP) and from CIDP+DSP.

**Methods:**

We performed a retrospective review of patients diagnosed with CIDP, DSP and CIDP+DSP (responsive to immunotherapy) and examined differences in CMAP duration and TD at baseline.

**Results:**

We included 59 subjects: 17 CIDP, 21 DSP and 21 CIDP+DSP. Of these, 16 (94.1%) CIDP, 18 (85.7%) CIDP+DSP and 1 (4.7%) DSP fulfilled the 2010 EFNS/PNS criteria for definite CIDP. There was no difference in CMAP duration or TD in all nerves (compound outcome) or in individual motor nerves. Patients with CIDP/CIDP+DSP had more conduction blocks, slower conduction velocities and more prolonged F wave latencies than those with DSP.

**Conclusion:**

Measures of CMAP duration and TD were not helpful in distinguishing CIDP, DSP or CIDP+DSP patients; however, parameters such as F-wave latencies, conduction blocks or the number of demyelinating parameters were useful in this separation.

**Significance:**

There are no definite nerve conduction criteria to distinguish patients with CIDP+DSP from DSP alone. Further studies focusing on measures of demyelination may provide stronger evidence to guide treatment decisions in CIDP + DSP patients.

## Introduction

Diabetic neuropathy is the most frequent complication of diabetes mellitus (DM), however the diagnosis is not always straightforward, as up to 50% of the patients can be asymptomatic (1). Clinical manifestations can be broad and non-specific, and include sensorimotor deficits, pain and autonomic dysfunction (1–3). Distal symmetric polyneuropathy (DSP) accounts for up to 50% of presentations in DM and includes symptoms of both large, myelinated nerve fibers (numbness, imbalance, reduced reflexes, proprioception/vibration sense) and small fibers (pain and reduced thermal discrimination) dysfunction (1, 4).

DSP usually affects the peripheral nervous system in a length-dependent fashion, where distal segments are predominantly involved, sensory nerves are damaged prior to motor nerves and rarely results in major weakness (3, 4). Changes to this usual pattern should prompt consideration of other causes such as acquired demyelinating neuropathies, that frequently affect motor more than sensory fibers in a non-length dependent fashion and have distinct electrophysiologic abnormalities (5, 6). Chronic inflammatory demyelinating polyneuropathy (CIDP) is the most common acquired chronic immune-mediated neuropathy, with prevalence ranging from 0.8 to 8.9 per 100,000 people (7). Some studies have found an increased prevalence of CIDP in DM patients and the coexistence of both diseases imposes significant diagnostic and therapeutic challenges (8–14). Furthermore, one recent study also showed a twofold increase in the risk of DM in two distinct CIDP European cohorts, also supporting this relationship (15).

The accurate diagnosis of CIDP in the context of DM should be a matter of utmost importance as CIDP is the most frequent treatable inflammatory neuropathy (9, 15–17). Various electrophysiologic assessments have been explored in prior studies, albeit no consensus has been reached regarding the best instrument to differentiate between CIDP and CIDP in association with DM. It has already been demonstrated that duration of the distal compound muscle action potential can differentiate patients with CIDP from chronic axonal and hereditary neuropathies (18–20). Motor, rather than sensory conduction studies, are more appropriate to distinguish the two conditions and are helpful to differentiate patients with diffuse slow or borderline motor CV and demyelinating features beyond what is expected for DSP (5, 21–24). Furthermore, the number of demyelinating features is associated with different treatment response rates in CIDP and CIDP+DM (22, 23). Interestingly, recent publications have shown that the compound muscle action potential (CMAP) duration and temporal dispersion (TD) did not differ between patients with CIDP treated with immunoglobulin versus placebo, suggesting that these parameters might be less sensitive to changes (25, 26).

The European Federation of Neurological Societies/Peripheral Nerve Society (EFNS/PNS) clinical and electrodiagnostic criteria for CIDP have been established as the most useful criteria in discriminating demyelinating neuropathies from other processes, demonstrating a very good balance between sensitivity and specificity (27). Among the demyelinating criteria, increased distal duration (or abnormal distal dispersion) of the CMAP is a well-established measure, and can be particularly helpful when other aspects of NCS show mild or borderline features of demyelination (18, 20, 28). In the updated EFNS/PNS criteria, new parameters for the distal CMAP duration based on the filters used have been established (29). Additional measures of temporal dispersion (TD), including an increase beyond 30% of CMAP duration from proximal to distal stimulation, are considered abnormal and included in the EFNS/PNS probable criteria (27). Abnormalities in F-waves may also be helpful in patients not fulfilling other electrodiagnostic criteria and also in those with poor tolerance for extensive studies (30).

The diagnosis and treatment of CIDP+DM is challenging and can be delayed, as symptoms might be erroneously attributed to DSP or other causes, which can lead to lack of treatment (31). Our primary objective was to investigate the potential contribution of NCS in discriminating CIDP from DSP and from CIDP+DSP by focusing on measures of duration and temporal dispersion (TD) of the distal CMAP.

## Methods

### Inclusion and Exclusion Criteria

We performed a retrospective review of patient charts previously coded as CIDP, DSP and CIDP+DSP from August 2007 - December 2020 according to expert opinion (VB) and included adult patients (aged 18 years and older) with a final diagnosis of DSP, CIDP and CIDP+DSP who responded to immunotherapy (as indicative of an autoimmune component as patients with DSP alone are unresponsive). To be included as a CIDP case, only typical variants were considered, defined as: (1) chronic, progressive, stepwise or recurrent, proximal and distal weakness/sensory dysfunction developing over at least 2 months and (2) reduced or absent reflexes in the upper and/or lower limbs. Patients with confirmed hereditary neuropathies, paraproteinemia, other autoimmune diseases, thyroid diseases and suspected paraneoplastic diseases were excluded. Patients had to be free of DM by the time of the first assessment.

DM diagnosis was confirmed according to the American Association of Diabetes criteria and was based on the following abnormalities: hemoglobin A1c, fasting plasma glucose, random elevated glucose with symptoms or abnormal 2-h oral glucose tolerance test (32). To be included as DSP, patients should have slowly progressive, predominantly distal sensory and/or sensorimotor symptoms involving lower or upper and lower limbs, with no significant proximal weakness and after exclusion of other common metabolic, toxic, infectious or paraneoplastic diseases. Patients with DM and phenotypes consistent with small fiber neuropathy, autonomic neuropathy, focal mononeuropathy (carpal tunnel syndrome, ulnar neuropathy at the elbow, fibular neuropathy), diabetic lumbosacral radiculoplexus neuropathy, diabetic cervical radiculoplexus neuropathy, thoracic radiculopathy, cranial neuropathy, were excluded. All patients with DSP fulfilled the Toronto Diabetic Neuropathy Expert Group criteria (4). Patients with DSP, who presented with progression or change in the pattern of neuropathy symptoms, areflexia and/or proximal weakness in a pattern consistent with CIDP were included as CIDP+DSP. In this situation, a lumbar puncture and/or MRI spine with gadolinium were requested for supportive diagnosis.

We extracted demographic data, physical examination, measures of disability, laboratory results and electrophysiologic data. Disability was measured by the RODS (Rasch-built Overall Disability Scale) in patients with CIDP and the ONLS (Overall Neuropathy Limitations Scale) in all patients (33, 34). The study protocol was approved by the University Health Network Research Ethics Board, based on chart review and collection of de-identified data.

### Electrophysiologic Studies

NCS were performed using the Sierra Wave instrument (Cadwell Laboratories Inc., Kennewick, WA). Age and height-adjusted reference values were used, according to the standards of the TGH (UHN) electrophysiology laboratory. Limb temperature was measured and maintained at ≥32.0°C in the hands and ≥31.0°C in the feet. Motor nerve CMAP amplitudes were measured as peak to trough with a sweep speed of 5 ms/div and a gain of 5 mv/div and the filters were 10-10k for all nerves. The F-wave latency was determined by both the minimum and maximum reproducible latency obtained after 10 supramaximal stimuli and using a sweep speed of 10 ms/div (and adjusted accordingly) and a gain of 500 v/div. Latencies, amplitudes and duration were measured for each nerve at each stimulus site and conduction velocity (CV) between stimulus sites. All motor tracings for the median, ulnar, fibular and tibial nerves were reviewed for quality assessment by two independent technicians (MN and JDC) and posteriorly EFNS/PNS guidelines were applied to classify each case (MN, MA and VB). We considered segments in the forearm and foreleg to determine the presence of demyelinating parameters. Conduction blocks were measured in the median, ulnar and fibular nerves. All NCS included in this study were done at baseline, prior to immunosuppressive/immunomodulating therapy in both CIDP and CIDP+DSP patients.

### Outcomes

Our main outcome was the difference in duration and TD of the distal CMAP duration between patients with CIDP, DSP and CIDP+DSP. CMAP duration was defined as the interval between onset of the first negative peak and return to baseline of the last negative peak for the following nerves as defined: median ≥ 6.6 ms, ulnar ≥ 6.7 ms, peroneal ≥ 7.6 ms and tibial ≥ 8.8 ms, according to EFNS/PNS guidelines (27, 28). This was scored as a compound outcome (median, ulnar, fibular and tibial nerves), with averaged values for each parameter (duration and TD). We hypothesized that as NCS are done in a standardized manner in our laboratory, using a compound measurement for each parameter, excessive demyelination would be apparent, and the groups could be reliably distinguished. Secondary outcomes were: the number of demyelinating parameters per patient; differences in amplitudes, CV and F-wave latency in individual nerves within the groups.

### Statistical Analysis

The data was assessed by plots and/or the Shapiro-Wilk test to verify any deviation from normal distributions. Demographic data were expressed as means/standard deviation (SD) for normally distributed data, or median and interquartile range (IQR) for the remainder. Differences in categorical variables were assessed using Fisher or Chi-squared tests, while differences in continuous variables (electrophysiologic parameters) were assessed by *t*-tests/Mann-Whitney; analysis of variance (ANOVA) or the Kruskal–Wallis test. Homogeneity of variances were assessed with Bartlett's test. Spearman's correlation coefficient was used to assess correlation between continuous variables. Results were adjusted for multiplicity with the Bonferroni correction and *p*-values < 0.05 were considered significant. In an exploratory analysis logistic regression was used to model the predicted probability of attaining definite or probable categories in the EFNS/PNS criteria according to the total number of demyelinating parameters. All analyses were performed in Stata version 16.1 (College Station, TX, USA).

## Results

We included 59 subjects: 17 with CIDP, 21 with DSP and 21 with CIDP+DSP. There was no difference in age, gender or the duration of the neuropathy in the groups or in the duration of DM in DSP and CIDP+DSP groups. Patients with CIDP/CIDP+DSP had greater disability when compared to patients with DSP (ONLS scale only). On the other hand, there were no differences in the level of disability between CIDP and CIDP+DSP patients (ONLS and RODS). There were no correlations between the level of disability with age or duration of neuropathy in all groups (Supplementary Table 1). The summary demographic data are shown in Table 1.

**Table 1:** Demographic and clinical profile of CIDP, DSP and CIDP + DSP patients.

	**CIDP 17 patients**	**DSP 21 patients**	**CIDP + DSP 21 patients**	***p*-value**
Age mean (SD); median; range	53.4 (9.5) 53 35–71	56.5 (15.9) 59 25–83	58.8 (8.9) 57 44–81	0.26
Sex (male) %	4 (23.53)	9 (42.86)	4 (19.05)	0.27
Duration of DM (years); mean (SD), range	NA	11.4 (2.3) (1–30)	11.7 (1.9) (0.5–27)	0.96
Duration of neuropathy (years) mean (SD); range	2.7 (2.5) 0.4–10.0	2.8 (2.4) 2.5–10.0	3.0 (3.1) 1–15.0	0.94
A1C mean (SD); range	NA	7.3 (0.4) 5.6–12	7.9 (0.9) 4.8–12	0.48
RODS	35.1 (8.9)	^*^	36.2 (7.9)	0.68
ONLS	2.2 (1.4)	1.7 (1.4)	3.1 (1.8)	**0.02**
Protein (CSF) mean (SD); range	1.0 (0.6) 0.63–2.2 (07 patients)	NA	1.2 (0.7) 0.47–2.36 (10 patients)	0.59

* *excluded from analysis as <50% with measurements; NA, not available. Bold values meant statistically significant*.

Sixteen CIDP patients (94.1%), 18 with CIDP+DSP (85.7%) and 1 with DSP (4.7%) fulfilled the EFNS/PNS electrodiagnosis criteria of definite disease before therapy (Table 2). Five patients with CIDP (29%), 6 with CIDP+DSP (28%) and 4 patients with DSP (19%) had abnormal TD. Nine patients with CIDP (52%), 10 with CIDP+DSP (47%) and 14 patients with DSP (66%) had abnormal distal CMAP duration. There was no difference in the duration or TD in the motor nerves in the groups, both in the compound primary outcome or in individual nerves (Table 2, Supplementary Table 1).

**Table 2:** Electrophysiologic criteria.

**EFNS/PNS Criteria**	**CIDP**	**DSP**	**CIDP + DSP**	***p*-value**
Definite (%)	16 (94.1)	1 (4.7)	18 (85.7)	<0.001^*^
Probable (%)	1 (5.9)	2 (9.5)	1 (4.8)	NA
Possible (%)	0 (0)	6 (28.6)	2 (9.5)	NA
No criteria (%)	0 (0)	12 (57%)	0 (0)	NA
Number of demyelinating parameters per individual, mean (SD), median, range	6.5 (3.3) 6 2–15	1.5 (1.5) 2 0–5	6 (2.5) 6 1–10	**<0.001**
Number of nerves with conduction block per patient, mean (SD), median, range	0.9 (0.7) 1 0–2	(0.3) 0 0–1	0.9 (0.9) 1 0–2	0.001^*^
Number of nerves with abnormal distal duration in each group, mean (SD), median, range	(1.2) 1 0–3	0.9 (0.6) 1 0–2	0.8 (0.8) 1 0–3	0.71
Number of nerves with abnormal TD, mean (SD), median, range	0.5 (0.8) 0 0–2	(0.3) 0 0–1	(0.6) 0 0–2	0.58
Duration (ms) in all nerves, mean (SD); median, IQR	6.9 (2.2) 6.2 5.5–7.4 (51 nerves)	7.5 (3.5) 6.1 5.3–8.4 (60 nerves)	6.9 (7.3) 6.1 5.7–6.6 (65 nerves)	0.64
TD (percentage prolongation from distal to proximal) in all nerves, mean (SD); median, IQR	22.3 (29.4) 13.5 6.3–27 (46 nerves)	16.7 (22.1) 12 5–21 (53 nerves)	15.1 (42.5) 8.5 2.5–20 (56 nerves)	0.14
Amplitude (mV) in all nerves, mean (SD); median, IQR	5.3 (3.4) 4.7 2.5–7.8 (51 nerves)	6.1 (4.0) 5.8 3.2–9.1 (60 nerves)	2.9 (3.0) 1.7 0.3–5.2 (65 nerves)	<0.001^**^
Conduction velocity (m/s) in all nerves, mean (SD); median, IQR	39.7 (8.2) 38 34–46 (49 nerves)	45.8 (9.4) 46 41–53 (58 nerves)	37.6 (8.7) 40 29–45 (58 nerves)	<0.001^*^
F-wave min (ms) in all nerves, mean (SD); median, IQR	51.9 (17.5) 48.4 36.4–68.4 (42 nerves)	41.0 (12.8) 36.9 27.5–49.1 (50 nerves)	42.3 (15.9) 35.5 32.6–40.9 (38 nerves)	<0.001^***^

* *both CIDP and CIDP+DSP showed significant differences when compared to DSP after adjustment*.

** *significant differences only for CIDP+DSP group (smaller amplitudes) when compared to other groups*.

*** *significant differences only for CIDP group (significantly prolonged F-waves) when compared to other groups. Bold values meant statistically significant*.

**Figure 1:**
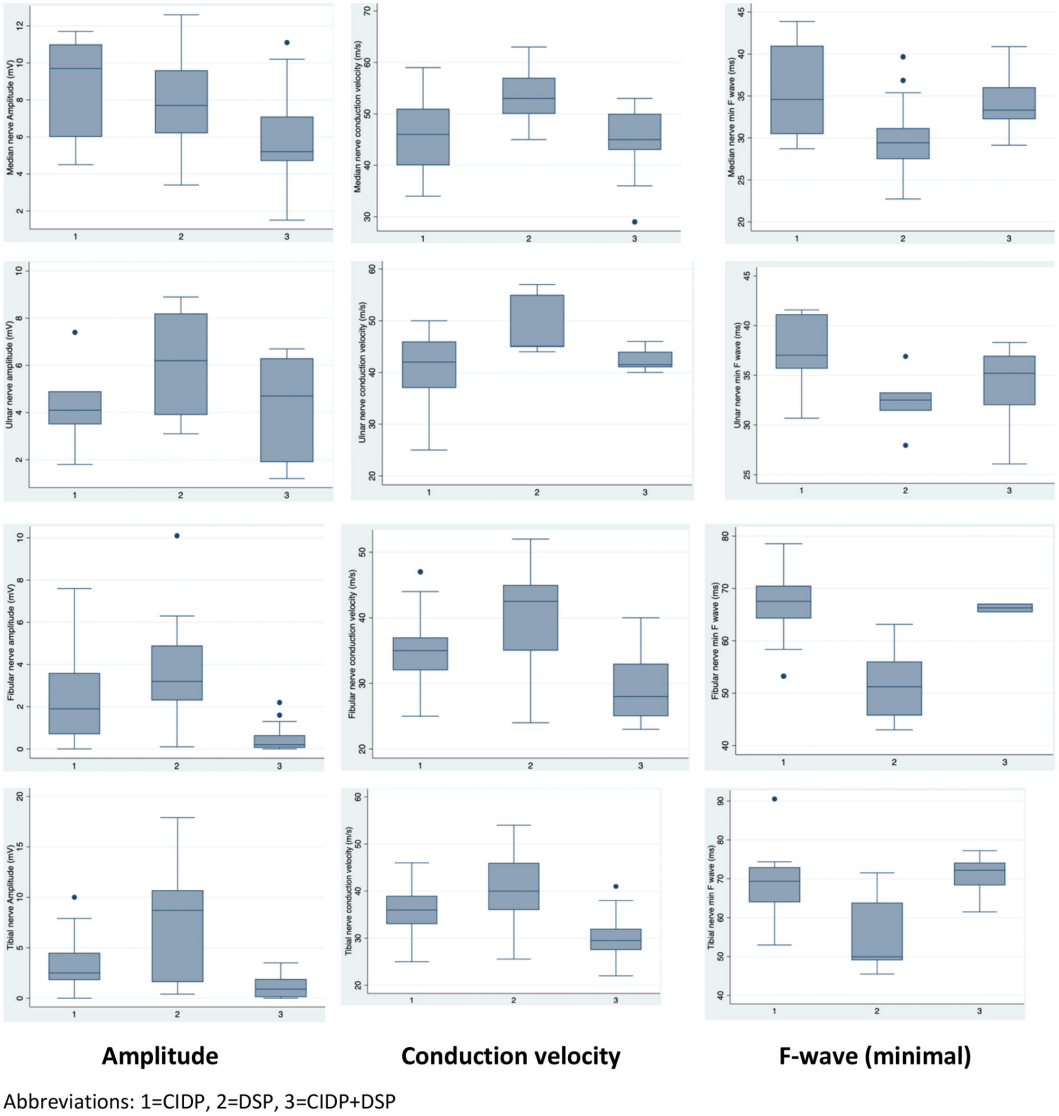
Electrophysiologic data by category.

Twelve CIDP patients (70.6%) and 13 CIDP+DSP patients (62%) had at least one nerve with CB. Three DSP patients (14.3%) had one nerve with CB and these nerves showed only ≥30% CMAP amplitude reduction. The criteria for ≥50% CMAP amplitude reduction was only fulfilled by CIDP patients (6 nerves) and CIDP +DSP (13 nerves). Patients with CIDP+DSP had significantly reduced amplitudes and prolonged F-waves when compared to other groups (Figure 1).

**Figure 2:**
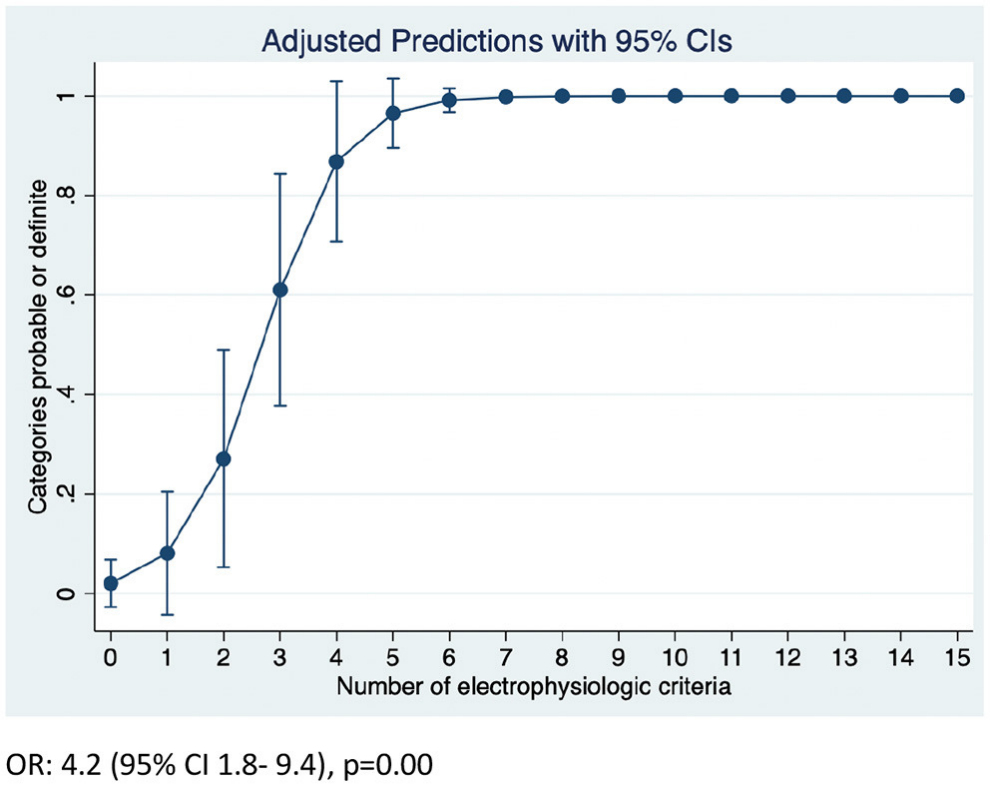
Predicted probability of attaining definite or probable categories according to EFNS/PNS criteria.

Overall, the number of demyelinating parameters (sum of number of nerves with either prolonged distal latency, increased duration, TD, reduced conduction velocity, CB or prolonged F waves) was higher in both CIDP and CIDP+DSP patients (Table 2). The predicted probability of attaining the EFNS/PNS definite or probable categories was close to 100% if 7 or more demyelinating features were present (95% CI 0.96–1.0) (Figure 2, Supplementary Figure 1). Furthermore, conduction velocities and F wave latencies showed significant differences in groups (all nerves combined) and individual nerves within the groups, with more demyelinating features in both CIDP and CIDP+DSP (Table 2, Figure 1, Supplementary Table 1). Using the updated EFNS/PNS criteria did not change the classification of our patients. In the ulnar and peroneal nerves, some individual parameters could not be compared due to limited observations (Supplementary Table 1).

## Discussion

There are multiple diagnostic challenges in differentiating CIDP from other neuropathies and one of the most important is the absence of a clear biomarker, which directly impacts treatment decisions. We have applied EFNS/PNS criteria in a systematic manner and while focusing on measures of distal CMAP duration and dispersion, we could not find any difference between the groups of CIDP, DSP or CIDP+DSP patients in our sample.

While unique parameters to distinguish demyelinating features in diseases that are predominantly axonal could provide invaluable support for the diagnosis of CIDP in the context of DM, this has been a matter of debate over the years and no consensus has been reached (5, 6, 8, 9). There are no clear-cut values of how much demyelination should be present in DM neuropathies to confirm a concomitant diagnosis of CIDP. In an exploratory analysis, we could demonstrate that patients with 7 or more demyelinating parameters attained the definite and probable EFNS/PNS criteria, which could indicate the patients that would be more likely responsive to therapy. In a previous work, it has been suggested that CIDP can be diagnosed in patients with DM when motor symptoms are more severe than expected and at least 3 of 4 electrophysiological criteria are fulfilled (reduced CV, CB/TD dispersion; increased distal motor latency and prolonged F-waves) (21). Although this is a matter of speculation, we consider that a more conservative approach and higher levels of certainty should be attained before offering a course of immunotherapy.

From a practical standpoint, the treatment response might be affected by the degree of demyelination. Some studies have provided evidence that fulfilling a higher number of electrophysiological criteria in CIDP patients, with and without DM, can be associated with better treatment responses (22, 23). Furthermore, it has already been demonstrated in clinical trials that the effects of the treatment in demyelinating parameters can be measured over short periods of time (25). Our study has provided evidence that patients with CIDP and CIDP+DSP fulfilled a higher number of demyelinating criteria (including more prolonged F-waves and reduced CV) as compared to DSP patients. Those measures, although not part of our primary outcome, could provide baseline parameters that could be compared in future follow-up studies in a larger sample.

Interestingly, we could also demonstrate that patients with DSP presented with less CB and their nerves showed CB only if defined as a ≥30% CMAP amplitude reduction, which would add only to “probable” category according to EFNS/PNS electrophysiologic criteria and would not be helpful in distinguishing categories. Furthermore, patients with CIDP and CIDP+DSP showed significantly reduced CV and patients with CIDP showed significantly prolonged F-wave latencies. Although this is also a matter of speculation, these findings suggest that as CV can be similarly reduced in both CIDP and CIDP+DSP patients, parameters such as CB and F-wave latencies might better differentiate CIDP patients from DSP. Additionally, F-waves were found to be more significantly prolonged only in CIDP patients, which could suggest a more diffuse demyelinating process, including more proximal segments, as compared to other groups. Whether the CB demonstrated in DSP patients could represent excess segmental demyelination in diabetic polyneuropathy and a superimposed process, is still a matter of debate and should be interpreted with caution.

The current study has several limitations. First, although only statistically significant and adjusted results were considered, our sample is small. Furthermore, misclassification and selection bias could be present, as there is no definitive biomarker to diagnose CIDP, either clinical or electrophysiologic.

In summary, measures of duration and dispersion of the distal CMAP were not helpful in distinguishing CIDP, DSP or CIDP+DSP patients in our sample. Our findings suggest that these parameters might be less useful than other variables (such as F-wave latencies or CB) to differentiate patients that could respond to therapy. Further studies focusing on other measures of demyelination might provide stronger evidence to guide treatment decisions in CIDP+DSP patients.

## Data Availability Statement

The original contributions presented in the study are included in the article/Supplementary Material, further inquiries can be directed to the corresponding author.

## Ethics Statement

The studies involving human participants were reviewed and approved by University Health Network Research Ethics Board. Written informed consent for participation was not required for this study in accordance with the national legislation and the institutional requirements.

## Author Contributions

MA contributed to data collection, statistical analysis, and first draft and review. MN and JC contributed to data collection and quality assessments. CB-T, HK, and VB contributed to design, analysis, and critical review. All authors contributed to the article and approved the submitted version.

## Conflict of Interest

The authors declare that the research was conducted in the absence of any commercial or financial relationships that could be construed as a potential conflict of interest.

## Publisher's Note

All claims expressed in this article are solely those of the authors and do not necessarily represent those of their affiliated organizations, or those of the publisher, the editors and the reviewers. Any product that may be evaluated in this article, or claim that may be made by its manufacturer, is not guaranteed or endorsed by the publisher.
