# Supplementary material for: Temporal Dispersion and Duration of the Distal Compound Muscle Action Potential Do Not Distinguish Diabetic Sensorimotor Polyneuropathy From Chronic Inflammatory Demyelinating Polyneuropathy
Source: Front Neurol. 2022 Apr 26;13:872762. doi: 10.3389/fneur.2022.872762 (PMC9087194; doi:10.3389/fneur.2022.872762)
Supplement: Supplementary file 1 [file Data_Sheet_1.pdf]

Supplementary Figure I - 95% CI intervals for the predicted probability of attaining definite or probable categories according to EFNS/PNS criteria

Number of demyelinating criteria	Delta-method Margin	Std. Err.	z	P>|z|	[95% Conf. Interval]	
1	.0204593	.0242319	0.84	0.398	-.0270343	.0679529
2	.0809254	.0631941	1.28	0.200	-.0429328	.2047836
3	.2707081	.1113309	2.43	0.015	.0525036	.4889127
4	.6101099	.1190035	5.13	0.000	.3768673	.8433524
5	.8683652	.0823279	10.55	0.000	.7070054	1.029725
6	.9652896	.0355126	27.18	0.000	.8956862	1.034893
7	.9915424	.0120486	82.30	0.000	.9679275	1.015157
8	.9979807	.0036836	270.93	0.000	.9907611	1.0052
9	.9995203	.0010673	936.50	0.000	.9974284	1.001612
10	.9998862	.0002991	3343.22	0.000	.9993	1.000472
