# Supplementary material for: Temporal Dispersion and Duration of the Distal Compound Muscle Action Potential Do Not Distinguish Diabetic Sensorimotor Polyneuropathy From Chronic Inflammatory Demyelinating Polyneuropathy
Source: Front Neurol. 2022 Apr 26;13:872762. doi: 10.3389/fneur.2022.872762 (PMC9087194; doi:10.3389/fneur.2022.872762)
Supplement: Supplementary file 2 [file Table_1.doc]

**Supplementary Table I - Nerve conduction study parameters for individual nerves**

| **Nerve** | **Measurement** | | **CIDP**  **mean (SD)** | **DSP (mean ± SD)** | **CIDP + DSP (mean ± SD)** | **p-value** |
| --- | --- | --- | --- | --- | --- | --- |
| **Median** | **dL(ms), mean (SD)** | | 4.7 (2.0) | 4.4 (1.25) | 4.6 (1.1) | 0.72 |
|  | **dCMAP amp (mV), mean (SD)** | | 8.6 (2.6) | 7.8 (2.4) | **5.8 (2.6)** | **<0.01** |
|  | **dCMAP dur (ms), mean (SD)** | | 6.2 (1.3) | 5.4 (0.9) | 6.1 (0.9) | 0.07 |
|  | **Conduction velocity (m/s), mean (SD)** | | **46.1 (7.4)** | 53.6 (5.5) | **45 (5.6)** | **0.00** |
|  | **TD p X d (%), mean (SD)** | | 14.6 (12.5) | 11.7 (14.2) | 14.3 (10.3) | 0.73 |
|  | **F min (ms), mean (SD)** | | **35.7 (5.7)** | 29.9 (4.2) | **33.7 (3)** | **<0.01** |
|  | **F min prolongation: number/absent (% within category)** | | 10 (58.2) | 2 (9.52) | 8 (40) | **<0.01** |
|  | **F-wave chronodispersion (ms), mean (SD)** | | **4.1 (2)** | 2.1 (1.2) | **4.1 (2.1)** | **<0.01** |
|  | **F-wave persistence, mean (SD)** | | 89.7 (14.6) | 98 (5.3) | 91.9 (8.4) | 0.05 |
|  | **% reduction in amplitude (proximal – distal)** | | 12.8 (12.9) | 5 (6.1) | 11.9 (18.4) | 0.16 |
|  | **Conduction block: number of nerves (% within category)** | | 3 (17.6) | 0 (0) | 4 (19.5) | 0.08 |
| **Nerve** | **Measurement** | | **CIDP**  **mean (SD)** | **DSP (mean ± SD)** | **CIDP + DSP (mean ± SD)** | **p-value** |
| **Ulnar** | **dL (ms), mean (SD)** | | 3.5 (0.9) | 2.6 (0.5) | 3 (0.3) | 0.05 |
|  | **dCMAP amp (mV), mean (SD)** | | 5.5 (1.5) | 6.1 (2.5) | 4.5 (2.2) | 0.33 |
|  | **dCMAP dur (ms), mean (SD)** | | 6.6 (0.7) | NA | 5.7 (0.7) |  |
|  | **Conduction velocity (m/s), mean (SD)** | | **40.3 (8.8)** | 49.2 (6.3) | **42.2 (2.1)** | **0.04** |
|  | **TD p X d (%), mean (SD)** | | 15.2 (16.6) | NA | 3.2 (7.1) |  |
|  | **F min (ms), mean (SD)** | | 37.2 (4.4) | 32.2 (3.2) | 33.9 (4.2) | 0.18 |
|  | **F min prolongation: number/absent (% within category)** | | 2 (40) | 4 (66) | 5 (50) | 0.21 |
|  | **F-wave persistence, mean (SD)** | | 92.6 (9) | 98 (2.6) | 97.7 (21.7) | 0.48 |
|  | **F chronodispersion (ms), mean (SD)** | | 3.4 (2) | 2.8 (2.1) | 3.9 (1.3) | 0.54 |
|  | **% reduction in amplitude (proximal – distal)** | | 23.1 (20.3) | 8.4 (3.7) | 9.4 (9.6) | 0.06 |
|  | **Conduction block: number of nerves (% within group)** | | 3 (50) | 0 (0) | 0 (0) | **0.03** |
| **Nerve** | **Measurement** | | **CIDP** | **DSP** | **CIDP + DSP** | **p-value** |
| **Fibular** | **dL (ms), mean (SD)** | | 5.9 (1.5) | 4.9 (2.2) | **7.2 (3.9)** | **0.002** |
|  | **dCMAP amp (mV), mean (SD)** | | 2.5 (2.2) | 2.3 (2.5) | **0.5 (0.6)** | **< 0.001** |
|  | **dCMAP dur (ms), mean (SD)** | | 7.5 (3.2) | 6.5 (1.4) | 5.7 (1.2) | 0.22 |
|  | **Conduction velocity (m/s), mean (SD)** | | **34.9 (6)** | 39.7 (8.1) | **29.7 (5.3)** | **0.000** |
|  | **TD p X d (%),mean (SD)** | | 19 (35.5) | 15.7 (28.8) | 16.4 (51.5) | 0.97 |
|  | **F min (ms), mean (SD)** | | **67 (7.4)** | 51.7 (6.7) | **66. 2 (1.1)** | **0.000** |
|  | **F min prolongation/absent : number (% within category)** | | 13 (86) | 0 | 12 (100) | **0.000** |
|  | **F-wave persistence, mean (SD)** | | 86.2 (12.1) | 89.4 (11.3) | NA | 0.67 |
|  | **F chronodispersion (ms), mean (SD)** | | 6.7 (3.2) | 4.6 (1.6) | NA | 0.07 |
|  | **% reduction in amplitude (proximal – distal)** | | 15.5 (26.7) | 6.9 (12.1) | 5.2 (26.7) | 0.55 |
|  | **Conduction block: number of nerves (% within group)** | | 3 (20) | 0 | 2 (13.3) | 0.08 |
| **Nerve** | **Measurement** | | **CIDP** | **DSP** | **CIDP + DSP** | **p-value** |
| **Tibial** | **dL (ms), mean (SD)** | | 5.7 (1.9) | 4.4 (1.1) | 5.6 (1) | 0.04 |
|  | **dCMAP amp (mV), mean (SD)** | | 3.5 (2.8) | 7.4 (5.9) | 1.1 (1.1) | **0.001** |
|  | **dCMAP dur (ms), mean (SD)** | | 7.1 (2.2) | 11.9 (4.1) | 10.3 (9.3) | 0.10 |
|  | **Conduction velocity (m/s), mean (SD)** | | **35.9 (5.5)** | 40.7 (8.0) | **30.3 (5.1)** | **0.00** |
|  | **TD p X d (%),mean (SD)** | | 39.3 (39.1) | 25.2 (20.1) | 23 (70.8) | 0.44 |
|  | **F min (ms), mean (SD)** | | **68.5 (9.5)** | 54.7 (9.0) | **70.6 (6.0)** | **0.001** |
|  | **F min prolongation: number (% within category)** | | 9 (81.8) | 2 (20) | 13 (92.8) | 0.00 |
|  | **F-wave persistence, mean (SD)** | | 87 (31.3) | 98.2 (3.9) | 87 (21) | 0.43 |
|  | **F chronodispersion (ms), mean (SD)** | | 5.3 (2.3) | 3.4 (1.7) | 6.7 (4.9) | 0.13 |
|  | **% reduction in amplitude (proximal – distal)** | | 12.8 (12.9) | 21.9 (12.8) | 29.0 (32.5) | 0.29 |
|  | **Conduction block: number of nerves (% within group)** | | 5.0 (41.6) | 1 (7.1) | 7 (77.8) | **0.002** |
| **Correlation matrix parameters** | **RODS** | | | **ONLS** | | |
| **Duration of neuropathy (p-value)** | CIDP  0.14 | DSP  NA | CIDP+DSP  1.0 | CIDP  0.33 | DSP  1.0 | CIDP+DSP  1.0 |
| **Correlation matrix parameters** | **Age** | | | **# demyelinating features** | | |
| **Duration of neuropathy (p-value)** | CIDP  0.77 | DSP  0.26 | CIDP+DSP  0.68 | CIDP  0.2 | DSP  0.93 | CIDP+DSP  0.59 |

Comments: temporal dispersion (TD) (%) prolongation distal -proximal. Where only 2 categories are present, t-tests or Mann-Whitney were used, otherwise ANOVA or Kruskal Wallis. NA- not available
